# Effect of Co-Diet Supplementation on Biodegradation of Polyethylene by *Galleria mellonella* L. (Lepidoptera: Pyralidae)

**DOI:** 10.3390/insects15090704

**Published:** 2024-09-16

**Authors:** Areej Mahfooz, Muhammad Yasin, Mirza Abdul Qayyum, Asim Abbasi, Abeer Hashem, Khalid F. Almutairi, Elsayed Fathi Abd_Allah, Muhammad Farhan, Muhammad Anjum Aqueel, Mishal Subhan

**Affiliations:** 1 Department of Entomology, Faculty of Agriculture and Environment, The Islamia University of Bahawalpur, Bahawalpur 63100, Pakistan; 2 Institute of Plant Protection, Muhammad Nawaz Sharif-University of Agriculture, Multan 66000, Pakistan; 3 Department of Entomology, University of Agriculture, Faisalabad 38040, Pakistan; asimuaf95@gmail.com; 4 Botany and Microbiology Department, College of Science, King Saud University, P.O. Box. 2460, Riyadh 11451, Saudi Arabia; 5 Plant Production Department, College of Food and Agricultural Sciences, King Saud University, P.O. Box. 2460, Riyadh 11451, Saudi Arabia; 6 College of Plant Protection, Yangzhou University, Yangzhou 225009, China; 7 Department of Microbiology and Molecular Genetics, The Women University Multan, Multan 66000, Pakistan

**Keywords:** plastic pollution, greater wax moth, polyethylene, biodegradation

## Abstract

**Simple Summary:**

This study investigates the biodegradation of polyethylene (PE) by *Galleria mellonella* larvae, exploring the impact of co-diet supplementation. Polyethylene, a prevalent plastic, poses significant environmental pollution due to its resistance to natural degradation. This research evaluates the larvae’s efficiency in consuming PE of various densities with and without co-diets like beeswax and wheat germ + honey. The findings indicate that the larvae consumed more PE when the density was lower, with 1 mm thickness showing the highest consumption and minimal weight loss when beeswax was added. The larvae demonstrated higher biodegradation rates within 24 h compared to within 48 and 72 h. The co-diet supplementation, particularly with beeswax, enhanced PE consumption, aligning with the larvae’s natural diet. This research underscores the potential of using *G. mellonella* larvae for the environmentally friendly biodegradation of PE, suggesting further exploration of the larvae’s digestive mechanisms to develop sustainable solutions for plastic pollution.

**Abstract:**

Pollution coming from plastic polymers, particularly polyethylene (PE), poses a serious threat to both humans and animals. The biodegradation of plastics facilitated by insects is a crucial and eco-friendly approach that can be employed to combat this global concern. Recently, the larvae of the greater wax moth *Galleria mellonella* (L.) have been recognized as avid ‘plastivores’. The current study was aimed at evaluating the feeding efficiency of *G. mellonella* larvae on PEs of various densities with a co-diet supplementation of wheat germ + honey and beeswax. The results reveal that maximum PE consumption (9.98 ± 1.25 mg) was recorded in the case of 1.0 mm thick PE after a 24 h interval; however, the same scenario also achieved the greatest reduction in larval weight (27.79 ± 2.02 mg). A significant reduction in PE mass (5.87 ± 1.44 mg) was also observed in 1.0 mm PE when fed beeswax; however, the larvae experienced minimal weight loss (9.59 ± 3.81 mg). The larvae exhibited a higher PE consumption in 1.0 mm PE, indicating that the lower the density of PE, the greater the consumed area. Moreover, the biodegradation levels were notably higher within the 24 h interval. In conclusion, these findings suggest that the density of PEs and the supplementation of the co-diet have an impact on PE biodegradation. Additionally, the utilization of *G. mellonella* for the biodegradation of PE proves effective when combined with beeswax, resulting in minimal weight loss of the larvae. Our findings offer initial insights into how *Galleria mellonella* larvae biodegrade polyethylene (PE) of four different densities, along with co-diet supplementation. This approach helps us evaluate how varying densities affect degradation rates and provides a better understanding of the larvae’s capabilities. Additionally, our observations at three specific time intervals (24, 48, and 72 h) allow us to identify the time required for achieving degradation rates. Through examining these time points, our method offers valuable insights into the initial phases of plastic consumption and biodegradation.

## 1. Introduction

The plastics industry has expanded significantly over the last seven decades, both in terms of production and utilization in nearly all aspects of our everyday life. This is because plastics have evolved exceptional characteristics over time, including affordability, stability, and durability [1,2,3]. Synthetic plastics are made from various hydrocarbons and petroleum derivatives and have a larger molecular weight because of small, repeated monomer units [4,5]. Among them, polyethylene (PE) alone makes up 64% of all plastics [6,7]. The plastics industry is expected to expand in the upcoming years, reaching an estimated value of about $1050 billion by 2033. According to current growth trends, the production of plastics is expected to double within the next twenty years [8,9]. 

Pollution from plastic waste is now widely recognized as a serious environmental problem. The main issue with plastics is that they are extremely long-lasting and can take hundreds of years to degrade [10,11]. However, only around half of all plastic waste produced has been either recycled or dumped in landfills [12]. A significant amount of the leftover plastic trash pollutes continents, oceans, and every part of the globe, transforming it into a ‘Plastic World’ [13,14]. During disposal, plastic debris release chemicals that are capable of leaching into the soil and contaminating the groundwater and soil [15,16]. The deposition of this debris in soil can lead to changes in drainage patterns, disruption of soil fauna, degradation of soil quality, and ultimately reduced agricultural yields. Moreover, microplastics (MPs) that are produced during plastic degradation have a size of less than 5 mm and have the potential to cause eco-toxicological effects [17,18,19,20]. Additionally, fibrous MPs have the potential to cause health impacts like carcinogenicity and mutagenicity because they can be breathed and may accumulate in the lungs [20,21].

Conventional plastic management approaches mainly rely on certain disposal methods mainly including landfilling, incineration, and recycling. In the early 2000s, the majority of plastic waste was managed through landfilling (65–70%) and incineration (20–25%), while only about 10% was recycled. However, this distribution varies by country, depending on factors such as the standard of living and population [22,23]. Despite these methods, a significant portion of plastic waste produced remains unattended. Moreover, landfills and incineration can lead to serious environmental problems through emission of hazardous pollutants, including dioxins, metals, carbon monoxide (CO), furans, and volatile organic compounds [24]. These ecological constraints make these approaches less effective in addressing this global issue [24,25].

In light of these problems, it is vital to seek environmentally friendly alternatives to tackle this issue, such as biodegradation [2,26]. Plastic biodegradation is an eco-friendly and cost-effective approach to combatting plastic pollution [27]. Biodegradation refers to the process of physical and chemical changes occurring within a substance because of the activity of microorganisms [28]. According to recent research, the larvae of the Greater Wax Moth, *Galleria mellonella* L. (Lepidoptera: Pyralidae), have been found to be capable of biodegrading low-density PEs [29,30]. *Galleria mellonella* is a serious insect pest of the beekeeping industry due to its destructive feeding habits by tunneling in the comb and feeding on pollen, honey, beeswax, and brood [31,32]. In addition to being a major pest in apiculture, its larvae can also feed on PE. Its larvae break down PE and convert it to ethylene glycol. PE degradation by *G. mellonella* larvae is attributed to the similar chemical structure of beeswax and PE. Beeswax is made of long-chain hydrocarbons, a complex mixture of lipid compounds that include alkanes, alkenes, fatty acids, and esters. As in PE, the most common hydrocarbon bond is CH_2_-CH_2_ [33,34]. The metabolic pathways involved in the degradation of long-chain hydrocarbons are expected to play an important role in the degradation of PE, which is composed of a long aliphatic chain [35]. Since *G. mellonella* larvae feed on and metabolizes long-chain hydrocarbons from beeswax, they may also potentially metabolize PE.

One hundred larvae of *G. mellonella* inflicted 92 mg weight loss in PE even after 12 h [33]. Gut enzymes and gut microbiota play a key role in PE degradation. The saliva of *G. mellonella* larvae contains two enzymes from the phenol oxidase family, demonstrating their capability to effectively degrade polyethylene [36]. Yang et al. [37] isolated two bacterial strains, *Enterobacter asburiae* YT1 and *Bacillus* sp. YP1, from the gut microbiota of *G. mellonella* larvae consuming PE. Approximately 6% and 11% of PE film was degraded by YT1 and YP1, respectively, after 60-day incubation. Likewise, different species of the genus *Enterobacter* and *Acinetobacter*, isolated from the guts of *G. mellonella*, were found to be degrading PE [38,39].

The current study is novel in terms of evaluating the feeding efficiency of *G. mellonella* larvae on polyethylene (PE) of four different densities (1 mm, 2 mm, 3 mm, and 4 mm) with and without co-diet supplementation (wheat germ plus honey and beeswax) at three specific time intervals (24, 48, and 72 h). Weight loss in both *G. mellonella* larvae and PE, reduction in PE surface area, and diet consumption were investigated. This study advances our understanding relating to the biodegradation potential of *G. mellonella* larvae across different PE densities and explores effective combinations of co-diet supplementation.

## 2. Materials and Methods

### 2.1. Polyethylene (PE) Bags

Polyethylene (PE) bags of four different densities (1, 2, 3, and 4 mm) were acquired from R.T. Plastic Agency, Bahawalpur, Punjab, Pakistan. Plastic pieces were cleaned with an air stream to eliminate any residues and later disinfected with 75% ethanol before being placed in a Petri dish [37,40].

### 2.2. Insect Collection and Rearing

Honey bee combs, naturally infested with *G. mellonella*, were obtained from the agricultural farm areas of the University of Agriculture Faisalabad, Pakistan, for the establishment of further colonies in the laboratory. The combs were brought to the Entomology Laboratory, Department of Entomology, Faculty of Agriculture and Environment, The Islamia University of Bahawalpur, Pakistan. They were transferred to plastic boxes (66 × 44 × 39 cm). The boxes’ lids were slightly modified by cutting a 2.5 cm diameter hole in the center and replacing it with ultra-thin stainless steel wire mesh (180 mesh size) for ventilation and to prevent small larvae from escaping. These larvae were reared on diet formulation described by de Jong et al. [32], derived from Hickin et al. [41], where rice flour was substituted for rice bran and wheat germ was substituted for wheat bran. The ingredients of the diet included beeswax (22 g), wheat germ (28 g), rice flour (54 g), oat meal (54 g), yeast (84 g), glycerol (128 g), honey (136 g), and water 960 (g) [32]. For larval diet preparation, all dry ingredients including beeswax, were mixed in a container. The liquid components were mixed in another beaker and heated in a microwave until lukewarm. The heated mixture was added to the dry ingredients and thoroughly mixed until no dry components remained visible [32]. The diet was offered to the larvae until pupation and adult emergence. Newly emerged adults were then shifted to a new plastic box for mating and egg laying. Pieces of hard white paper were hung in the box to provide females a substrate for egg laying. After every 48 h, the papers with cluster of eggs were removed and replaced with new papers. The rearing conditions involved maintaining complete darkness at 27.0 ± 2.0 °C and 60.0 ± 5.0% relative humidity (r.h.) in an incubator [32].

### 2.3. Weight Loss in G. mellonella Larvae and PE, Reduction in PE Surface Area, and Diet Consumption

Ten homogenous larvae weighing 90 ± 0.3 mg were taken from the culture collection. The larvae were deprived of food for 36 h to avoid the effects of previously consumed food. A bioassay was performed in plastic Petri dishes (100 mm), with PEs measuring 5 cm^2^ in size. Twelve different feeding conditions were provided to the larvae (Table 1). The one-gram (g) diet, larvae, and PEs were weighed prior to experimentation, and the Petri dishes were placed in an incubator under the above-mentioned conditions. The data regarding diet consumption, PE, larval weight loss, and surface area reduction were collected 24, 48, and 72 h after release. The graph paper method was used to measure the surface area of the holes made on the PE [42], and a digital weighing balance (Shimadzu ATX224 Analytical balance from 0.1 mg to 220 g capacity) was used to assess the changes in weight of the plastics, larvae, and diet. The whole experiment was replicated thrice.

### 2.4. Statistical Analysis 

The data regarding larval weight loss, surface area reduction, and polyethylene weight loss from the trials were analyzed through a two-way analysis of variance (ANOVA), wherein the treatments and exposure interval served as the main effect with larval weight loss, surface area reduction, and PE weight loss as the response variables. The analysis considered interactions among all main effects. The assessment of diet consumption in the combined treatments of PE + (wheat germ and honey) and PE + beeswax involved a two-way ANOVA, with treatments and exposure intervals taken as the main effects, and the diet consumption taken as the response variable. Interactions between the main effects were also incorporated into the analysis. To make comparisons in larval weight loss, surface area reduction, PE weight loss, and diet consumption means were separated for significance using the Tukey–Kramer test (HSD) at a 5% significance level [43]. Statistical analysis was performed using Minitab, 2017 [44].

## 3. Results

### 3.1. Weight Loss in G. mellonella Larvae and PE and Reduction in PE Surface Area

Significant differences were observed in the main effects and interaction for surface area reduction, while interaction effects on larvae and PE weight loss were non-significant (Table 2). Larval weight reduction was observed across all the treatments and exposure intervals. The highest weight loss was observed in the sole treatment of PE_1_, followed by PE_2_, PE_3_, and PE_4_. Overall, larval weight loss tended to be higher in PE treatments compared to the combination of PE with wheat germ and honey, or beeswax. 

Significant differences in the weight loss of *G. mellonella* larvae were observed across all the treatments and exposure intervals (Table 3). After 24 h of exposure, significantly more larval weight loss was observed primarily in larvae fed solely on PEs (PE_1_, PE_2_, PE_3_, and PE_4_). PE_1_ exhibited the highest larval weight loss (27.79 ± 2.02 mg), followed by PE_2_, PE_3_, and PE_4_ in sole treatments. In combined treatments, the highest larval weight loss was recorded in PE_1_ + WgH (19.69 ± 2.88 mg), followed by PE_2_ + WgH, PE_3_ + WgH, and PE_4_ + WgH, while the minimum was recorded in PE_4_ + BW (4.91 ± 4.44 mg), followed by PE_3_ + BW, PE_2_ + BW, and PE_1_ + BW. A similar trend was observed at 48 h of exposure, with the highest weight loss recorded for PE_1_ treatment (6.79 ± 1.84 mg). The minimum weight loss was observed for PE_4_ + BW (3.9 ± 3.46 mg). After 72 h, PE_1_ still exhibited significantly more weight loss (7.53 ± 1.44 mg) compared to all other treatments, while PE_4_ + BW resulted in minimal weight loss (3.08 ± 4.04 mg). Throughout all exposure intervals, larvae subjected to PE-only treatments exhibited significantly higher weight loss than those in combined treatments. Notably, the combination treatments involving PEs + BW did not result in more than 19.33 mg of larval weight loss. During the exposure period of 24 h, the larval weight loss was highest compared to the 48 and 72 h exposure intervals (Table 3).

Regarding surface area reduction, significant differences were observed in the surface area of PE consumed by larvae in all the treatments and exposure intervals (Table 4). After 24 h of exposure, a notable reduction in surface area was recorded in PE alone (PE_1_, PE_2_, PE_3_, and PE_4_), particularly in PE_1_ (5 ± 0.59 cm^2^), followed by PE_2_, PE_3_, and PE_4_ in sole treatments. Among the combined treatments, PE_1_ + BW resulted in the highest surface area reduction (3.25 ± 0.57 cm²), followed by PE_2_ + BW, PE_3_ + BW, and PE_4_ + BW. The lowest reduction was recorded for PE_4_ + WgH (0.5 ± 0.04 cm²), followed by PE_3_ + WgH, PE_2_ + WgH, and PE_1_ + WgH. A similar trend was observed at 48 h of exposure, with PE_1_ showing the highest surface area reduction (1.5 ± 0.57 cm²). The lowest reduction was noted for PE_4_ + WgH (1.0 ± 0.75 cm²). After 72 h, PE_1_ exhibited significant surface area reduction (3.5 ± 0.60 cm^2^) compared to all other treatments, while PE_4_ + WgH resulted in the minimal surface area reduction (0.5 ± 0.28 cm^2^). Throughout all exposure intervals, PE-only treatments showed significantly greater surface area reduction than the combined treatments. Notably, combined treatments involving PEs + WgH did not result in more than a 3.25 cm² reduction in surface area. During the exposure period of 24 h, the surface area reduction was highest compared to the 48 and 72 h (Table 4). 

Significant differences in PE weight loss were observed across all the treatments and exposure intervals (Table 5). After 24 h of exposure, significant weight loss of PE was observed when larvae were exposed to PEs alone treatments (PE_1_, PE_2_, PE_3_, and PE_4_), particularly in PE_1_ (9.98 ± 1.25 mg), followed by PE_2_, PE_3_, and PE_4_. In combined treatments, highest PE weight loss was recorded in PE_1_ + BW (5.87 ± 1.44 mg), followed by PE_2_ + BW, PE_3_ + BW, and PE_4_ + BW, while minimum was recorded in PE_4_ + WgH (1.29 ± 3.23 mg), followed by PE_3_ + WgH, PE_2_ + WgH, and PE_1_ + WgH. Likewise, after 48 h, with PE_1_ showing the maximum PE weight loss (4.01± 1.15 mg). The lowest reduction was noted for PE_4_ + WgH (1.1 ± 3.04 mg). After 72 h, PE_1_ exhibited significant PE weight loss (6.5 ± 0.57 mg) compared to all other treatments, while PE_4_ + WgH resulted in the minimal PE weight loss (3.1 ± 2.82 mg). Throughout all exposure intervals, single PE treatments resulted in significantly higher PE weight loss compared to the combined treatments (Table 5). 

### 3.2. Diet Consumption (Wheat Germ and Honey) by G. mellonella Larvae

Regarding diet consumption, the main effects were found to be significant, while their interaction effects were non-significant (Table 6). Significant differences were observed in the diet consumption of beeswax when provided with PEs of various densities in all the treatments and exposure intervals.

**Figure 1:**
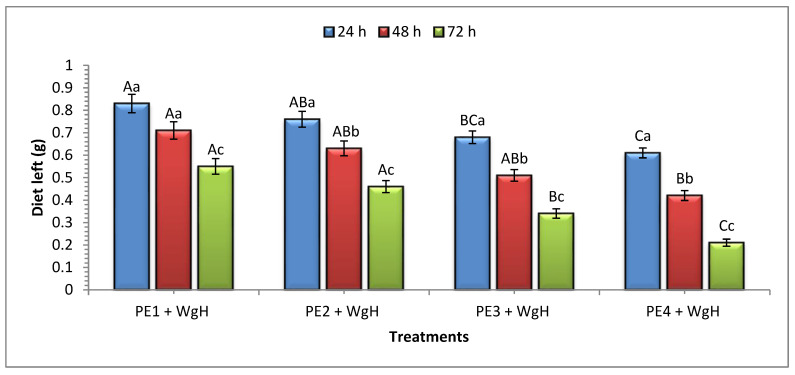
Mean (±SE) consumption (g) of wheat germ + honey mixture provided with PEs of different densities at 24, 48, and 72 h of exposure intervals. For each exposure interval (24 h, *F* = 20.3; df = 3, 11; *p* ≤ 0.01; 48 h, *F* = 5.63; df =3, 11; *p* ≤ 0.01; 72 h, *F* = 48.4; df =3, 11; *p* ≤ 0.01), means followed by the same uppercase letter are not significantly different (Tukey–Kramer (HSD) test at *p* = 0.05). For treatments (PE_1_ + WgH, *F* = 127; df = 2, 8; *p* ≤ 0.01; PE_2_ + WgH, *F* = 120; df =2, 8; *p* ≤ 0.01; PE_3_ + WgH, *F* = 35.1; df =2, 8; p ≤ 0.01; PE_4_ + WgH, *F* = 120; df =2, 8; p ≤ 0.01), means followed by the same lowercase letter are not significantly different (Tukey–Kramer (HSD) test at *p* = 0.05). PE_1_ + WgH: PE 1 mm + WG + H; PE_2_ + WgH: PE 2 mm + WG + H; EP_3_ + WgH: PE 3 mm + WG + H; PE_4_ + WgH: PE 4 mm + WG + H. The initial weight of WgH was 1 g.

Significant differences were observed in the diet consumption of the WgH when provided with PEs of various densities across all the treatments and exposure intervals (Figure 1). After 24 h of exposure, significant consumption of WgH was recorded in PE_4_ + WgH (0.39 ± 0.02 g) compared to all other treatments, followed by PE_3_ + WgH (0.32 ± 0.01 g), and PE_2_ + WgH (0.24 ± 0.02 g), while the minimum was observed in PE_1_ + WgH (0.17 ± 0.03 g). A similar trend in diet consumption was observed after 48 h, with the maximum diet consumption in PE_4_ + WgH (0.19 g) and the minimum consumption in PE_1_ + WgH (0.12 g). After 72 h, PE_4_ + WgH exhibited the highest consumption (0.21 g), while PE_1_ + WgH resulted in the minimum consumption (0.16 g). During the exposure period of 24 h, the consumption of WgH was the highest compared to the 48 and 72 h intervals (Figure 1).

### 3.3. Diet Consumption (Beeswax) by G. mellonella Larvae

**Figure 2:**
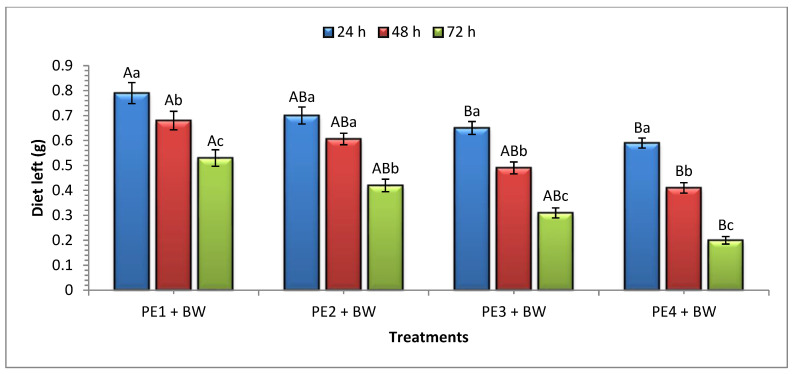
Mean (±SE) consumption (g) of beeswax provided with PEs of different densities at 24, 48, and 72 h exposure intervals. For each exposure interval (24 h, *F* = 8.18; df = 3, 11; *p* ≤ 0.01; 48 h, *F* = 4.88; df =3, 11; *p* ≤ 0.01; 72 h, *F* = 6.56; df =3, 11; *p* ≤ 0.01), means followed by the same uppercase letter are not significantly different (Tukey–Kramer (HSD) test at *p* = 0.05). For treatments (PE_1_ + WgH, *F* = 110; df = 2, 8; *p* ≤ 0.01; PE_2_ + WgH, *F* = 35.8; df =2, 8; *p* ≤ 0.01; PE_3_ + WgH, *F* = 25.0; df =2, 8; p ≤ 0.01; PE_4_ + WgH, *F* = 52; df =2, 8; p ≤ 0.01), means followed by the same lowercase letter are not significantly different (Tukey–Kramer (HSD) test at *p* = 0.05). PE_1_ + WgH: PE 1 mm + WG + H; PE_2_ + WgH: PE 2 mm + WG + H; EP_3_ + WgH: PE 3 mm + WG + H; PE_4_ + WgH: PE 4 mm + WG + H. The initial weight of BW was 1 g.

Significant differences were observed in the consumption of beeswax when provided with PEs of various thicknesses in all the treatments and exposure intervals (Figure 2). After 24 h of exposure, significantly more consumption of beeswax was recorded in PE_4_ + BW (0.41 ± 0.01 g), compared to all other treatments, followed by PE_3_ + BW (0.35 ± 0.02 g), and PE_2_ + BW (0.3 ± 0.01 g), while the minimum was observed in PE_1_ + BW (0.21 ± 0.03 g). A similar trend in consumption was observed after 48 h, with the maximum consumption in PE_4_ + BW (0.18 g) and the minimum in PE_1_ + BW (0.11 g). After 72 h, PE_4_ + BW exhibited the highest diet consumption (0.21 g), while PE_1_ + BW resulted in the minimum consumption (0.15 g). Overall, the maximum diet consumption was recorded at 24 h of exposure compared to the 48 and 72 h (Figure 2).

## 4. Discussion

Polyethylene is the most widely used synthetic polymer globally. However, its resistance to natural degradation has led to its accumulation in landfills and the environment [45,46,47]. Consequently, there is a significant need to identify effective methods to expedite its biodegradation process, which has been a focal point of extensive research in recent years. Recently, the larvae of the *G. mellonella*, a significant pest in the bee industry, have been recognized as ‘plastivores’ [48,49]. These larvae exhibit the remarkable ability to consume and metabolize PE [50]. In our study, we used PEs of different densities alone and in combination with beeswax and wheat germ + honey against the larvae of the *G. mellonella*. The results show that the highest weight loss of PE and larvae was observed when larvae were provided with PE alone. A significant reduction in the PE mass was also observed when larvae fed on PE in combination with beeswax, while the larvae experienced minimal weight loss. The larvae exhibited a higher PE consumption with a thickness of 1 mm, indicating that the smaller the density of PE, the greater the consumed area, while the lowest consumption of PE was with a density of 4 mm. The biodegradation level was notably higher at 24 h.

The biodegradation of PE could be attributed to the gut microbiota of *G. mellonella* larvae, which contain bacteria and fungi from various genera. Noel et al. [51] observed that the addition of low-density polyethylene does not significantly alter the bacterial microbiota of *G. mellonella* larvae at either the community or taxonomic levels, indicating the resilience of the bacterial microbiome. Similarly, Yang et al. [14] isolated two bacterial strains, *Enterobacter asburiae* YT1 and *Bacillus* sp. YP1, from the gut microbiota of *G. mellonella* larvae consuming PE. Likewise, bacteria from genus *Acinetobacter* and *Enterobacter* sp. D1, were isolated from the guts of *G. mellonella*, possessing the ability to degrade PE [33,34]. On the other hand, the fungus *Aspergillus flavus* was also found to degrade high-density MP particles into low-molecular-weight MP particles [52]. Researchers also accept that the saliva of *G. mellonella* larvae exhibits the ability to oxidize and depolymerize polyethylene. Within the saliva, two enzymes from the phenol oxidase family have been identified, demonstrating their capability to effectively degrade polyethylene [36].

In our study, when examining the consumption rate on the first day of the experiment, it was observed that ten larvae on 1 mm plastic (PE_1_) consumed 9.98 mg of PE, resulting in a larval weight loss of 27.29 mg. In comparison, 5.87 mg of PE was consumed in combination with beeswax (PE_1_ + BW), leading to a larval weight loss of 9.59 mg in 24 h. In a comparable study by Bombelli et al. [33], it was noted that 100 *G. mellonella* larvae could biodegrade 92 mg of polyethylene in 12 h. Despite extending the duration to 24 h in our study, we utilized only 10 larvae and achieved a greater amount of biodegraded PE. Alkassab et al. [53] reported that 10 larvae of *G. mellonella* could degrade 0.0210 mg in 12 h at 25 °C. Mandal and Vishwakarma [54] demonstrated that 480 larvae of the *G. mellonella*, conditioned at a temperature of 27 °C biodegraded 0.173 mg of PE over a period of 7 days. In their study, the biodegradation efficiency was lower compared to the findings in our research, even though the treatment duration was longer. They also noted that there was no correlation between relative humidity and larval growth.

However, our research revealed that *G. mellonella* larvae exhibited a higher biodegradation rate of low-density PE at 24 h compared to 48 and 72 h. This difference could be attributed to the notable number of larvae in Petri dishes at 24 h, where they actively fed. This behavior differed from the observations at 48 and 72 h when larvae were dispersed and did not consume PE in the same manner. Kwadha et al. [55] noted that larvae tend to feed more when they are in close proximity. Additionally, the larvae observed at 24 h were more active compared to those at 48 and 72 h, as they were entering the pupation stage [56]. In comparison to these studies, our research demonstrated better results in terms of weight loss in low density polyethylene (LDPE) biodegradation, and the duration of the study was relatively shorter. Additionally, temperature emerged as a significant factor influencing LDPE biodegradation. Alkassab et al. [53] conducted experiments with *G. mellonella* larvae at 25 °C and 35 °C, highlighting that the optimal temperature for biodegradation is 25 °C. According to Rodríguez Vega [56], *G. mellonella* larvae exhibit normal development at a temperature of 25 °C, with a lifespan of around 28 days. Deviations from this temperature can affect their activity and lifespan: lower temperatures slow down activity and prolong lifespan, while higher temperatures accelerate activity and significantly shorten lifespan. Consequently, our research was conducted at a temperature of 25 °C.

In our study involving the co-diet supplementation of *G. mellonella*, we employed beeswax, wheat germ, and honey. In previous studies, co-diet supplementation with beeswax resulted in a higher PE consumption [54,57]. This aligns with our findings, as the treatment with beeswax yielded better results, possibly because under natural conditions, these larvae typically feed on beeswax, and their bodies are accustomed to it [55]. Furthermore, it was observed that the growth of *G. mellonella* was unaffected by the ingestion of polyethylene because the composition and chemical structure of LDPE closely resemble those of beeswax [33]. For future research projects, evaluations of the pupation rate and survival of tested insects could offer additional insights into the ecology of insect species when exposed to polyethylene (PE). Investigating these factors would improve our understanding of how PE affects both pupation and survival rates of tested insects. 

Decisively, it has become an established fact that using insects to degrade plastics does not create secondary pollution, and these insects can latterly also be used as poultry and fish feed, providing both practical and economic benefits. However, using insects for plastic degradation is still an emerging field, and the current findings are not yet ready for large-scale application. Future research should focus on high-throughput sequencing to analyze the microbes in the insects’ guts, understanding how they break down plastics, and discovering new ways to improve this process. Moreover, policymakers must support the integration of researcher from diverse fields such as the environment and poultry and fish industries to obtain greater and sustainable benefits via insect rearing on plastics. 

## 5. Conclusions 

The consumption and degradation of plastics by insect larvae offers a novel approach for addressing plastic pollution. Our research concluded that the density of plastic and the addition of a co-diet significantly influence plastic biodegradation. Moreover, the use of *G. mellonella* for PE biodegradation is notably effective when conditioned with beeswax because of minimal larvae weight loss. The plastic feeding rate was significantly higher in 1 mm plastic compared to 2 mm, 3 mm, and 4 mm, indicating a decrease in larval consumption with an increase in plastic thickness. Within a confined environment, under suitable temperature conditions, it appears promising to employ these larvae to feed on plastic waste. However, further research is recommended to understand the intricate mechanisms within their digestive processes. Uncovering and replicating such mechanisms artificially holds the potential to provide a solution to the persistent issue of plastic pollution.

## Figures and Tables

**Table 1:** Treatments applied alone and in combination with wheat germ and beeswax.

Treatments	Composition
PE_1_	Polyethylene 1.0 mm
PE_2_	Polyethylene 2.0 mm
PE_3_	Polyethylene 3.0 mm
PE_4_	Polyethylene 4.0 mm
PE_1_ + WgH	Polyethylene 1.0 mm + Wheat germ and Honey
PE_2_ + WgH	Polyethylene 2.0 mm + Wheat germ and Honey
PE_3_ + WgH	Polyethylene 3.0 mm + Wheat germ and Honey
PE_4_ + WgH	Polyethylene 4.0 mm + Wheat germ and Honey
PE_1_ + BW	Polyethylene 1.0 mm + Beeswax
PE_2_ + BW	Polyethylene 2.0 mm + Beeswax
PE_3_ + BW	Polyethylene 3.0 mm + Beeswax
PE_4_ + BW	Polyethylene 4.0 mm + Beeswax

**Table 2:** ANOVA parameters for larval weight loss, surface area reduction, and polyethylene weight loss in laboratory experiment (total df = 107).

Effect	Df	Larval Weight Loss		Surface Area Reduction		PE Weight Loss	
		F	*p*	F	*p*	F	*p*
Treatment	11	75.20	≤0.05	76.29	≤0.05	412.33	≤0.05
Time	2	118.89	≤0.05	215.12	≤0.05	108.51	≤0.05
Treatment × Time	22	0.56	0.93	2.91	≤0.05	0.62	0.8947

**Table 3:** Mean (±SE) weight loss (mg) in *Galleria mellonella* larvae after 24, 48, and 72 h after exposure fed on PE alone and different combinations of co-diets. For each treatment, within each row, means followed by the same uppercase letter are not significantly different (Tukey–Kramer (HSD) test at *p* = 0.05). For each treatment, within each column, means followed by the same lowercase letter are not significantly different (Tukey–Kramer (HSD) test at *p* = 0.05).

Treatments	Final Larval Weight (mg)				
	24 h	48 h	72 h	F_2_,_8_	*p*
PE_1_	62.21 ± 2.02 D a	55.42 ± 1.84 F b	47.89 ± 1.44 F c	48.4	≤0.05
PE_2_	65.24 ± 2.19 CD a	58.31 ± 2.02 EF b	51.67 ± 1.73 EF c	34.9	≤0.05
PE_3_	67.32 ± 2.48 B–D a	61.43 ± 2.13 DEF b	53.93 ± 2.02 D–F c	27.4	≤0.05
PE_4_	68.65 ± 2.59 A–D a	63.12 ± 2.25 C–F a	55.12 ± 2.30 D–F b	24.4	≤0.05
PE_1_ + WgH	70.31 ± 2.88 A–D a	66.31 ± 2.30 B–F ab	60.12 ± 2.59 C–F b	11.7	≤0.05
PE_2_ + WgH	73.67 ± 3.17 A–D a	69.24 ± 2.42 A–E ab	62.32 ± 2.88 B–F b	12.2	≤0.05
PE_3_ + WgH	76.35 ± 3.46 A–D a	71.52 ± 2.59 A–E ab	66.52 ± 3.06 A–E b	7.75	≤0.05
PE_4_ + WgH	77.58 ± 3.75 A–D a	72.15 ± 2.77 A–D ab	68.04 ± 3.23 A–D b	6.41	≤0.05
PE_1_ + BW	80.41 ± 3.81 A–C a	75.42 ± 2.88 A–C ab	70.67 ± 3.46 A–C b	6.14	≤0.05
PE_2_ + BW	81.63 ± 3.92 A–C a	77.12 ± 3.06 AB ab	71.32 ± 3.63 A–C b	6.34	≤0.05
PE_3_ + BW	83.25 ± 4.04 AB a	79.63 ± 3.17 AB a	76.15 ± 3.81 AB a	2.77	0.1402
PE_4_ + BW	85.09 ± 4.44 A a	81.19 ± 3.46 A a	78.11 ± 4.04 A a	2.29	0.1820
F_11,35_	5.17	10.3	11.1		
*p*	≤0.05	≤0.05	≤0.05		

PE_1_: PE 1 mm; PE_2_: PE 2 mm; PE_3_: PE 3 mm; PE_4_: PE 4 mm; PE_1_ + WgH: PE 1 mm + WG + H; PE_2_ + WgH: PE 2 mm + WG + H; PE_3_ + WgH: PE 3 mm + WG + H; PE_4_ + WgH: PE 4 mm + WG + H; PE_1_ + BW: PE 1 mm + beeswax; PE_2_ + BW: PE 2 mm + beeswax; PE_3_ + BW: PE 3 mm + beeswax; and PE_4_ + BW: PE 4 mm + beeswax. Mean initial larvae weight, 90 mg.

**Table 4:** Mean (±SE) reduction in PE surface area (cm^2^) alone and in combinations with co-diets after 24, 48, and 72 h. For each treatment, within each row, means followed by the same uppercase letter are not significantly different (Tukey–Kramer (HSD) test at *p* = 0.05). For each treatment, within each column, means followed by the same lowercase letter are not significantly different (Tukey–Kramer (HSD) test at *p* = 0.05).

Treatments	Final Surface Area (cm^2^)				
	24 h	48 h	72 h	F_2_,_8_	*p*
PE_1_	20.00 ± 0.59 F a	18.50 ± 0.57 C b	15.00 ± 0.60 E c	57.4	≤0.05
PE_2_	20.50 ± 0.28 F a	19.00 ± 0.69 BC b	16.50 ± 0.71 DE c	34.7	≤0.05
PE_3_	21.00 ± 0.34 EF a	20.00 ± 0.80 A–C a	17.50 ± 0.75 DE b	22.2	≤0.05
PE_4_	21.50 ± 0.23 DEF a	20.50 ± 0.75 A–C a	18.00 ± 1.03 C–E b	17.4	≤0.05
PE_1_ + WgH	23.75 ± 0.57 AB a	22.75 ± 0.92 AB ab	21.75 ± 0.75 A–C b	5.19	≤0.05
PE_2_ + WgH	24.00 ± 0.28 AB a	23.00 ± 0.55 AB ab	22.00 ± 0.57 AB b	12.8	≤0.05
PE_3_ + WgH	24.25 ± 0.02 AB a	23.15 ± 1.06 AB a	22.75 ± 0.46 A a	4.07	0.0765
PE_4_ + WgH	24.50 ± 0.04 A a	23.50 ± 0.75 A ab	23.00 ± 0.28 A b	8.17	≤0.05
PE_1_ + BW	21.75 ± 0.57 C–F a	20.25 ± 1.15 A–C a	17.00 ± 0.98 DE b	20.3	≤0.05
PE_2_ + BW	22.50 ± 0.28 B–E a	21.00 ± 0.92 A–C a	18.50 ± 0.86 B–E b	22.1	≤0.05
PE_3_ + BW	23.00 ± 0.23 A–D a	22.00 ± 0.86 A–C a	19.50 ± 0.92 A–D b	17.8	≤0.05
PE_4_ + BW	23.50 ± 0.17 A–C a	22.50 ± 0.57 A–C a	20.00 ± 0.57 A–D b	43.1	≤0.05
F_11,35_	18.9	4.24	12.8		
*p*	≤0.05	≤0.05	≤0.05		

PE_1_: PE 1 mm; PE_2_: PE 2 mm; PE_3_: PE 3 mm; PE_4_: PE 4 mm; PE_1_ + WgH: PE 1 mm + WG + H; PE_2_ + WgH: PE 2 mm + WG + H; PE_3_ + WgH: PE 3 mm + WG + H; PE_4_ + WgH: PE 4 mm + WG + H; PE_1_ + BW: PE 1 mm + beeswax; PE_2_ + BW: PE 2 mm + beeswax; PE_3_ + BW: PE 3 mm + beeswax; and PE_4_ + BW: PE 4 mm + beeswax. Initial surface area, 25 cm^2^.

**Table 5:** Mean (±SE) weight loss (mg) in PE alone and in combination with co-diets after 24, 48, and 72 h. For each treatment, within each row, means followed by the same uppercase letter are not significantly different (Tukey–Kramer (HSD) test at *p* = 0.05). For each treatment, within each column, means followed by the same lowercase letter are not significantly different (Tukey–Kramer (HSD) test at *p* = 0.05).

Treatments	Final Weight of PE (mg)				
	24 h	48 h	72 h	F_2_,_8_	*p*
PE_1_	21.12 ± 1.25 G a	17.11 ± 1.15 I b	10.61 ± 0.57 I c	78.9	≤0.05
PE_2_	31.65 ± 1.44 EF a	27.85 ± 1.73 GH b	22.35 ± 1.27 GH c	29.5	≤0.05
PE_3_	44.01 ± 2.05 CD a	40.99 ± 2.02 D–F ab	35.97 ± 2.54 D–F b	10.1	≤0.05
PE_4_	57.38 ± 2.79 AB a	54.41 ± 2.51 A–C ab	49.91 ± 2.88 A–C b	5.69	≤0.05
PE_1_ + WgH	29.21 ± 1.27 E–G a	27.13 ± 1.15 GH a	23.05 ± 1.73 GH b	14.9	≤0.05
PE_2_ + WgH	37.97 ± 1.38 DE a	35.91 ± 1.78 E–G ab	31.92 ± 2.25 E–G b	8.40	≤0.05
PE_3_ + WgH	50.63 ± 2.50 BC a	49.54 ± 2.21 B–D a	46.14 ± 2.65 B–D a	2.72	0.1444
PE_4_ + WgH	63.11 ± 3.23 A a	62.01 ± 3.04 A a	58.91 ± 2.82 A a	1.55	0.2875
PE_1_ + BW	25.23 ± 1.44 FG a	21.19 ± 1.38 HI b	15.69 ± 0.86 HI c	43.7	≤0.05
PE_2_ + BW	35.88 ± 1.73 DE a	32.08 ± 1.90 FG a	27.00 ± 2.16 F–H b	15.9	≤0.05
PE_3_ + BW	48.03 ± 2.52 C a	44.93 ± 2.36 C–E ab	39.96 ± 2.77 C–E b	7.61	≤0.05
PE_4_ + BW	60.79 ± 3.01 A a	57.92 ± 2.96 AB a	53.52 ± 3.00 AB a	4.50	0.0640
F_11,35_	65.9	59.1	46.3		
*p*	≤0.05	≤0.05	≤0.05		

PE_1_: PE 1 mm; PE_2_: PE 2 mm; PE_3_: PE 3 mm; PE_4_: PE 4 mm; PE_1_ + WgH: PE 1 mm + WG + H; PE_2_ + WgH: PE 2 mm + WG + H; PE_3_ + WgH: PE 3 mm + WG + H; PE_4_ + WgH: PE 4 mm + WG + H; PE_1_ + BW: PE 1 mm + beeswax; PE_2_ + BW: PE 2 mm + beeswax; PE_3_ + BW: PE 3 mm + beeswax; and PE_4_ + BW: PE 4 mm + beeswax. Initial PE weights: 1 mm (31.1 mg), 2 mm (40.6 mg), 3 mm (52.2 mg), and 4 mm (64.4 mg).

**Table 6:** ANOVA parameters for co-diet consumption in laboratory experiment (total df = 35).

Effect	Df	Consumption of WgH		Consumption of Beeswax	
		F	*p*	F	*p*
Treatment	3	122.39	≤0.05	60.98	≤0.05
Time	2	290.96	≤0.05	155.48	≤0.05
Treatment × Time	6	1.95	0.1137	1.47	0.2310

## Data Availability

The data are available in the manuscript. Further inquiries can be directed to the corresponding authors.
